# Simultaneous Determination of Black Tea-Derived Catechins and Theaflavins in Tissues of Tea Consuming Animals Using Ultra-Performance Liquid-Chromatography Tandem Mass Spectrometry

**DOI:** 10.1371/journal.pone.0163498

**Published:** 2016-10-03

**Authors:** Souradipta Ganguly, Taposh Kumar G., Sudarshan Mantha, Koustubh Panda

**Affiliations:** 1 Department of Biotechnology and Guha Centre for Genetic Engineering & Biotechnology, University of Calcutta, Kolkata, India; 2 Waters Application Lab, Waters India Private Limited, Bangalore, India; National Institutes of Health, UNITED STATES

## Abstract

The bioavailability, tissue distribution and metabolic fate of the major tea polyphenols, catechins and theaflavins as well as their gallated derivatives are yet to be precisely elucidated on a single identification platform for assessment of their relative bioefficacy *in vivo*. This is primarily due to the lack of suitable analytical tools for their simultaneous determination especially in an *in vivo* setting, which continues to constrain the evaluation of their relative health beneficiary potential and therefore prospective therapeutic application. Herein, we report a rapid and sensitive Ultra-Performance Liquid Chromatography Tandem Mass Spectrometry (UPLC-MS/MS) based method for the simultaneous determination of the major catechins and theaflavins in black tea infusions as well as in different vital tissues and body fluids of tea-consuming guinea pigs. This method allowed efficient separation of all polyphenols within seven minutes of chromatographic run and had a lower limit of quantification (LLOQ) of ~5 ng/ml. Using this method, almost all bioactive catechins and theaflavins could be simultaneously detected in the plasma of guinea pigs orally administered 5% black tea for 14 days. Our method could further detect the majority of these polyphenols in the lung and kidney as well as identify the major catechin metabolites in the urine of the tea-consuming animals. Overall, our study presents a novel tool for simultaneous detection and quantitation of both catechins and theaflavins in a single detection platform that could potentially enable precise elucidation of their relative bioavailability and bioefficacy as well as true health beneficiary potential *in vivo*. Such information would ultimately facilitate the accurate designing of therapeutic strategies utilizing high efficacy formulations of tea polyphenols for effective mitigation of oxidative damage and inflammation in humans as well as prevention of associated diseases.

## Introduction

Tea is one of the most popular beverages consumed worldwide [1, 2] of which black tea accounts for about 80% of the global tea consumption [3]. It is a rich source of dietary polyphenols, that include different catechins i.e. epigallocatechin (EGC), epicatechin (EC), epicatechin-3-gallate (ECG), epigallocatechin-3-gallate (EGCG) and various theaflavins including theaflavin (TF), theaflavin-3-monogallate (TF3G), theaflavin-3'-monogallate (TF3'G) and theaflavin-3,3'-digallate (TF33'diG) (S1 Fig). The health benefits of tea consumption, especially with respect to its potential to prevent and mitigate cancer, cardiovascular diseases, diabetes, lung emphysema and several other degenerative diseases are well documented and extensively reviewed [1, 2, 4–7]. These health benefits have been attributed mainly to the antioxidant, anti-proliferative and anti-inflammatory effects of the tea polyphenols [2, 7–9]. Such revelations have triggered the use of the individual tea polyphenols or tea extracts enriched in these polyphenols as potential preventive against diseases associated with oxidative damage.

Despite such growing interests, the precise bioavailability, tissue distribution and the metabolic fate of these polyphenols remain undetermined, mostly due to the lack of a rapid and sensitive analytical tool for their simultaneous detection in different tissues [10, 11]. Attributing the exact health benefits associated with tea consumption to specific tea polyphenols thus remains largely unaccomplished. The lack of pharmacokinetic information related to the assimilation of tea polyphenols in various biological tissues and organs also makes it particularly difficult to recommend effective dosages of polyphenols for designing and conducting physiologically relevant clinical studies [4, 7]. This perhaps explains why several epidemiological studies have generated conflicting evidences on the predicted clinical benefits of tea consumption [12–15]. Prospective human trials using individual or combination of tea polyphenols thus cannot be accurately designed without relevant animal data revealing the precise bioavailability, tissue distribution, and bioefficacy of such tea polyphenols [10].

Several methods for analytical determination of tea polyphenols in biological samples have been developed. Most of these analyses have been used for catechins, while only a few for theaflavins [3]. The conventional and typically used method for separation and detection of tea polyphenols has been High-Performance Liquid Chromatography (HPLC) deploying either ultraviolet-visible spectrophotometric (UV/VIS) [16–18], electrochemical (ECD) [19–21], or chemiluminescence [22] detection. However, these methods suffer from one or more constraints including lack of adequate sensitivity (UV/VIS detection), the requirement for several post-separation processing steps (chemiluminescence detection) and protracted run times (applicable for all the above detection methods) which apparently limit their overall acceptability. The recent development of soft ionization methods, especially electrospray ionization (ESI) using tandem mass-spectrometric (MS/MS) interfacing, has significantly improved sensitivity and selectivity in such detection [3, 23, 24]. Coupling such mass spectrometric (MS) detection to an ultra-performance liquid chromatography (UPLC) based separation platform considerably reduces separation time and therefore solvent consumption, operational costs and analytical errors besides improving the overall sensitivity and selectivity of detection [25, 26].

Notwithstanding such reforms, most of the existing MS-based methods are incapable of simultaneously detecting the two major classes of bioactive tea polyphenols namely, catechins and theaflavins. This is largely because such kind of analyses require periodic switching between the positive and the negative polarity during electrospray ionisation (ESI) of the analytes, in order to enable detection of catechins in negative mode and theaflavins in positive mode, within the same cycle of detection. Indeed, simultaneous detection of catechins and theaflavins can provide pertinent information regarding their relative bioavailability and bioefficacy, while obviating the additional cost, time, labour and systemic errors associated with separately running two independent methods for achieving this goal. Further, most of the existing methods for catechin analyses are dedicated to the measurement of total catechins, which include their bioactive non-conjugated free forms as well as their largely bio-inactive conjugated forms. This profoundly restricts necessary derivation of relevant information on the actual bioefficacy of the physiologically important free polyphenols and identification of their metabolites [17, 27].

Our present endeavour was to develop a rapid and sensitive UPLC/MS-MS based method that can simultaneously determine catechins and theaflavins not only in different varieties of black tea preparations but also in various biological tissues including plasma, lung, kidney, liver and heart of animals (guinea pigs) orally administered black tea. To accomplish simultaneous detection of the eight major catechins and theaflavins, we resolved to use tandem mass spectrometry with positive/negative polarity switching during such measurement. Such measure allowed the effective acquisition of multiple reaction monitoring (MRM) transition mass spectra, in both ionization modes, from a single LC-MS/MS analysis. This also resulted in the development of the first UPLC-MS/MS based method for simultaneous detection of catechins and theaflavins that could not only help in the determination of the precise bioefficacy and bioavailability of such bioactive polyphenols in different biological tissues and organs *in vivo*, but also in the potential epidemiological and clinical evaluation of the proper dosage of these polyphenols required to formulate high efficacy tea products for effective prevention of oxidative damage and inflammation in humans.

## Materials and Methods

### Chemicals & Reagents

All tea polyphenol standards used were purchased from Sigma-Aldrich, USA. The Assam and Darjeeling black tea leaves were purchased from Goodricke Group Ltd. an internationally reputed tea merchant. Every experiment described herein was conducted with tea of the same lot for both the Darjeeling and Assam black tea. All other reagents used for the study were procured from Merck, Germany. A stock solution of the polyphenol standards (each 1 mg/ml) was prepared with 0.2% ascorbic acid and 0.005% EDTA and stored in small aliquots at -80°C till further use.

### Preparation of Tea Infusions

0.5 g of Assam and Darjeeling varieties of black tea were separately added to 10 ml of boiling water and brewed for 5 min with continuous stirring before being filtered into a hermetically sealed container [6]. The filtrates were designated as the Assam Black Tea (ABT) infusion and Darjeeling Black Tea (DBT) infusion respectively.

### Treatment of Animals

Male short-hair guinea pigs weighing 350–450 g were used for our experiments. All animal treatment procedures were carried out in strict accordance with the recommendations of the Guide for the Care and Use of Laboratory Animals of the National Institutes of Health (NIH), USA. The treatment protocol was approved by the Institutional Animal Ethics Committee (IAEC), Department of Biochemistry, University of Calcutta [Permit Number: IAEC/CU/BIOCHEM/DJC-KP (5)]. Throughout the animal treatment, all efforts were made to minimize stress and suffering of the animals. The guinea pigs were administered a polyphenol free diet (wheat bran 700 g, casein 200 g, sucrose 80 g, USP XVII salt mixture 10 g, AOAC vitamin mixture 10 g/kg of feed) and was orally administered water *ad libitum* along with suboptimal levels of vitamin C (2 mg/kg animal body weight) from seven days prior to the initiation of the experiment, to prevent scurvy [6]. The animals were finally divided into the following experimental groups (6 animals per group); ***Control Group*:** Orally administered water (20 ml/kg body wt) instead of black tea in two equal dosages daily for 14 days; ***Experimental Group I*:** Orally administered 20 ml/kg body weight of 5% Assam Black Tea (ABT) infusion in two equal dosages daily for a period of 14 days; ***Experimental Group II*:** Orally administered 20 ml/kg body weight of 5% Darjeeling Black Tea (DBT) infusion in two equal dosages daily for a period of 14 days. The time interval between the two dosages was maintained at 4 hours with the tea infusions freshly prepared before each administration. After being fed with the polyphenol-free diet for seven days, the guinea pigs belonging to the two experimental groups described above, were orally administered either ABT or DBT infusion for additional 14 days after which they were euthanized through intravenous ketamine hydrochloride injection around 120 min after the final oral administration of tea infusion (6 hours after the first dose and 2 hours after the second tea dose, on the 14^th^ day of treatment). Blood was immediately collected in vacutainer tubes containing ethylene-diamine-tetraacetate (EDTA) as anticoagulant and was centrifuged at 1000 g for 15 min at 4°C for the separation of plasma. The plasma was collected carefully and immediately mixed with an ascorbate-EDTA solution (20% ascorbic acid and 0.1% EDTA (w/v) in 0.4 M NaH_2_PO_4_ buffer, pH 3.6) in the ratio of 10:1 (plasma: ascorbate-EDTA solution) and stored at -80°C for subsequent use. The lung, kidney, liver and heart tissues were excised and collected from the experimental animals, washed in cold saline solution and stored at -80°C for further use. The urine was directly collected from the urinary bladders of the animals during their sacrifice, mixed with relevant volume of ascorbate-EDTA buffer (pH 5.0) and then stored at -80°C for subsequent use.

### Preparation of Samples for Analyses

Polyphenols were extracted from plasma and organs by the liquid-liquid extraction method described by Lee *et*. *al*. with slight modifications [19]. 200 μl of the thawed plasma was mixed with 20 μl of ascorbate-EDTA solution and 20 μl of phosphate buffer (pH 7.4). To this, 2.0 ml of ethyl acetate was added, vortexed for 5 min and centrifuged at 4500 rpm for 20 minutes. The upper ethyl acetate layer was then collected and after a second extraction, the combined supernatant was evaporated under nitrogen at 35°C to dryness. The dried sample was reconstituted with 100 μl of 15% acetonitrile containing 0.2% acetic acid. This was centrifuged at 13,000 rpm for 10 min, and 30 μl of the supernatant was loaded in the UPLC auto-sampler for relevant analysis of polyphenols.

For processing the tissue samples, 500 mg of lung, kidney, liver and heart tissues were homogenised using a mechanical homogenizer in 2.5 ml of the ascorbate-EDTA solution. The tissue lysates were then precipitated with 200 μl of ethanol to which 500 μl of dichloromethane was added. The mixture was then vortexed and centrifuged at 16,000 g for 5 min. The upper aqueous phase was collected, and after a second extraction with water, the pooled aqueous phase was again extracted with ethyl acetate similar to that done with the plasma samples. The spiked plasma and organ lysates were prepared by adding definite amounts of authentic standard mixture of polyphenols to the blank plasma and organ lysates collected from the control guinea pigs that were orally administered water instead of tea infusion, and were similarly processed by ethyl acetate extraction along with their blanks.

For identification of metabolites in the urine, 200 μl of the collected urine was centrifuged and the supernatant was diluted with equal volume of reconstitution solution consisting of 15% acetonitrile and 0.2% acetic acid and thereafter used for metabolite analyses.

### Instrumentation

Liquid chromatography (LC) coupled with tandem quadrupole mass spectrometry (LC/MS/MS) and operated in ‘multiple reaction monitoring’ (MRM) mode, was used for determination of catechin and theaflavin levels in our study, considering the potential of this technique to accomplish high selectivity and sensitivity in quantitative bioanalysis.

The liquid chromatography (LC) system consisted of a binary solvent delivery system comprising of two pumps and a refrigerated auto-sampler with fixed-loop injector (Acquity Binary UPLC system, Waters, USA). Chromatographic conditions were standardized through several trials to achieve best possible sensitivity and resolution by efficient separation of the analytes from possible matrix interferences. The chromatography was optimized on an Atlantis T3 (4.6 mm x 50 mm x 3 μm) C18 column (Waters, USA) that was maintained at 40°C. Separation was achieved using a mobile phase consisting of (A) aqueous solution of 0.2% acetic acid and (B) acetonitrile containing 0.2% acetic acid, at a flow rate of 0.8 ml/min. For analysis, 30 μl of the sample was injected under a programmed gradient which was initiated with 98% A, then ramped down to 70% A within 4 min, and was maintained at 70% A up to 7 min, before being changed to 2% A by 7.10 min, in order to accomplish efficient separation of the polyphenols. This was kept unchanged till 8 min following which the system was re-equilibrated for a fresh run.

A tandem quadrupole mass spectrometer, Xevo-TQD MS system (Waters, USA) with the capability of electrospray ionisation (ESI) switching between positive and negative polarity conditions was used. ESI negative mode was used for catechins while positive mode was used for detection of theaflavins. The capillary voltage was maintained at 3 KV, cone voltage at 30 V, and extractor voltage at 4 V for both positive and negative mode to achieve the best sensitivity for the analytes. The source temperature and desolvation temperatures were maintained at 150°C and 625°C, respectively. The cone gas flow rate was kept at 50 lit/hr and that of the desolvation gas flow at 1100 lit/hr. Catechins and theaflavins were identified based on their respective retention times as well as MRM transitions. The metabolites in urine were studied by precursor ion scan by monitoring the parent ions based on an earlier report [27].

### Software Used and Statistical Analysis

Data acquisition was performed with MassLynx 4.1 from Waters Corporation, USA, and was processed with TargetLynx application manager. The MassLynx software is commercially and globally available with UPLC systems coupled to Waters Xevo TQD Mass Spectrometers as acquisition control software, while TargetLynx is deployed for the purpose of quantitation of the acquired data. Certain statistical analyses were also performed with Origin Pro 8, scientific graphing and data analysis software from Origin Lab Corp. USA.

Regression analysis was used to evaluate the linearity of the developed method for individual catechins and theaflavins, and also to estimate their concentrations in the unknown samples. The paired t-test (α = 0.05) was used to determine whether the observed difference in the levels of the catechins and theaflavins between the 5% Assam black tea (ABT) and the Darjeeling black tea (DBT) infusions as well as the plasma and tissue lysates of the animals administered water against tea infusions, was statistically significant. The same test was also used to determine the significance of difference between the polyphenol levels detected in the lung and kidney lysates of animals orally administered black tea infusions.

## Results and Discussion

### Method Development

Our preliminary analysis, which included detection of tea polyphenols separately in positive and negative electro-spray ionisation (ESI) modes, indicated that catechins were best detected in negative mode whereas theaflavins in positive mode. This permitted higher selectivity in detection without compromising the detection sensitivity and hence could render the simultaneous detection of catechins and theaflavins possible in complex tissue matrices. This also mandated the use of an effective mass-spectrometric (MS) system that could accomplish equally efficient detection of both catechins and theaflavins despite accommodating the required switching between the two polarities in a single run. We were precisely able to accomplish this using the Xevo-TQD MS triple quadrupole mass spectrometer (Waters, USA) which is capable of switching between positive and negative polarities within 20 milli seconds. The data acquisition in MS/MS mode was optimized for the best possible selectivity and sensitivity for each analyte with collision energy of 20 for TF, TF3G and ECG, 18 for TF33'diG and EGC, 16 for EC and 15 for EGCG. Both the quadrupoles were operated at unit resolution for such runs. The MRM transitions which showed the highest response and signal-to-noise (s/n) ratio for each analyte were 565 to 139 for TF, 717 to 139 for TF3G; 869 to 139 and 869 to 744 for TF33'diG, 289 to 245 and 289 to 179 for EC, 305 to 125, 305 to 139 and 305 to 179 for EGC, 441 to 289 and 441 to 169 for ECG and 457 to 169 for EGCG. Other parameters, like those related to the nebulizer and the heater gases, were optimized to obtain a better spray shape for improved ionization. For the extraction of the polyphenols, a simple and widely used liquid-liquid extraction technique was employed as described under the ‘Materials and Methods’ section [19].

The present method was initially developed with the aqueous standards of the polyphenols and was subsequently modified for the matrix extracted samples. The aqueous linearity of the method, which was examined by running mixtures of polyphenol standards from 5 to 320 ng/ml, (EC analyzed from 10–640 ng/ml), showed linear and reproducible curves, with correlation coefficients of 0.996119, 0.9920, 0.998915, 0.999207 respectively for EGC, EC, EGCG, and ECG and 0.998375, 0.996340, 0.997688, 0.988960 for TF, TF3G, TF3'G, TF33'diG. The precision and accuracy of the method, determined by running six injections of different quality control standards, showed that they were well within the acceptable limits [28, 29] (Table 1). Table 1 also delineates the limit of detection (LOD), the lower limit of quantification (LLOQ), correlation coefficient (r) and linear regression (LR) of our developed method. Such aqueous linearity data was used to determine the relative content of polyphenols in the ABT and DBT infusions (S2 Fig). For achieving improved accuracy in the simultaneous detection and quantification of the polyphenols, we determined detection and quantification limits in consonance with the Clinical and Laboratory Standards Institute (CLSI) EP17 and the US-FDA guidelines while taking into consideration several parameters including the blank response, LOB (limit of blank), accuracy-precision as well as % R.S.D (percentage relative standard deviation) of analyte responses at low concentrations [28–30]. A uniform LLOQ (lower limit of quantification) of ~5 ng/ml was determined for all the polyphenols across all the analyzed matrices which helped us to accomplish a signal to noise (s/n) ratio well above the conventional s/n ratio of 1:10 (S1 Table).

**Table 1:** Precision (R.S.D in %), Accuracy (%), Limit of Detection (LOD), the Lower Limit of Quantification (LLOQ), Linear Regression (LR) and Correlation Coefficient (r), for the Analyzed Catechins and Theaflavins^1^.

Analyte	R.S.D (%)^2^^,^^3^		Accuracy (%)^3^	LOD (ng/ml)^2^^,^^4^	LLOQ (ng/ml)^2^^,^^5^	Linear Regression (LR)^2^	Correlation Coefficient (r)^2^
	Retention Time (RT)	Concentration					
**EGC**	0.10	5.36	109.9	0.77	5	Y = 54.55727X+315.40192	0.99799
**EC**	0.11	11.22	112.8	2.55	10	Y = 10.12788X+318.24962	0.99112
**EGCG**	0.11	8.21	108.4	1.03	5	Y = 140.07053X+236.47232	0.99907
**ECG**	0.12	4.85	103.5	0.74	5	Y = 219.8157X+728.55521	0.99916
**TF**	0.14	13.70	96.4	1.44	5	Y = 48.6907X–193.32261	0.99972
**TF 3 G**	0.22	9.79	118.1	2.56	5	Y = 56.77961X–171.11963	0.99996
**TF 3′ G**	0.06	8.33	104.5	2.70	5	Y = 45.48141X–200.1644	0.99961
**TF 3 3′ diG**	0.06	11.94	105.4	3.43	5	Y = 20.78482X–141.00132	0.99953

EGC, Epigallocatechin; EC, Epicatechin; EGCG, Epigallocatechin-3-gallate; ECG, Epicatechin-3-gallate; TF, Theaflavin; TF3G, Theaflavin-3-monogallate; TF3'G, Theaflavin-3'-monogallate; TF33'diG, Theaflavin-3,3'-digallate

^1^Method was developed with aqueous standards of the analyzed polyphenols.

^2^R.S.D, Relative Standard Deviation; LOD, limit of detection; LLOQ, lower limit of quantification; LR, Linear Regression; r, Correlation Coefficient

^3^Six analyses were performed for each polyphenol at LLOQs of 5 ng/ml or 10 ng/ml and concentrations of 20 ng/ml, 175 ng/ml and 320 ng/ml

^4^LOD = LOB + 1.645 (SD low concentration sample); where LOB is the Limit of Blank

^5^LLOQ refers to the lowest concentration on the calibration curve which can be quantitatively determined with precision and accuracy appropriate to the analyte and matrix. This typically had a response at least 5 times that of the blank.

**Fig 1:**
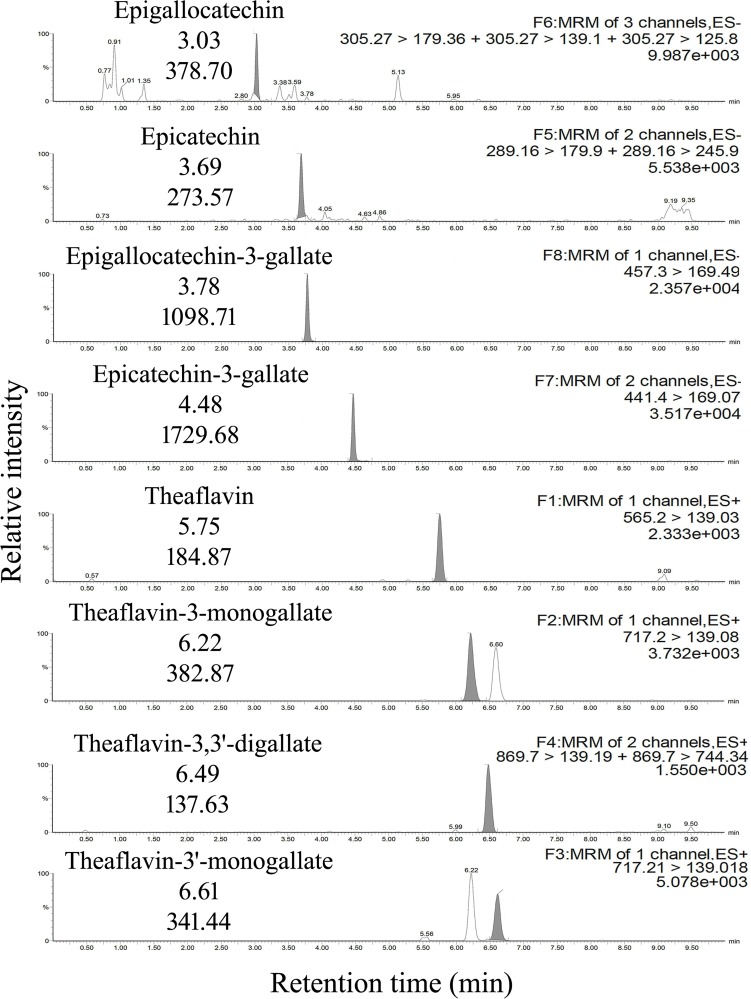
Detection of Catechins and Theaflavins in Plasma Spiked with Individual Polyphenols. Representative MRM chromatograms of epigallocatechin (EGC), epicatechin (EC), epigallocatechin-3-gallate (EGCG), epicatechin-3-gallate (ECG), theaflavin (TF), theaflavin-3-monogallate (TF3G), theaflavin-3,3'-digallate (TF33'diG), theaflavin-3'-monogallate (TF3'G), in blank plasma (control) spiked with the polyphenol standards to the final concentration of their LLOQ, showing the retention time (labelled below the analyte name) and response (labelled below the retention time) of the analytes (analyte names highlighted against their corresponding chromatograms). The right-hand top labels on the chromatograms indicate their respective MRM transitions and peak heights. Data are representative of three independent experiments done under similar conditions.

The linearity, sensitivity and selectivity of the method with respect to different biological matrices were initially determined through analyses of the plasma and organ lysates spiked with individual polyphenols. Such linearity data was subsequently used for quantifying catechins and theaflavins in the tissues of tea consuming guinea pigs (Fig 1, S3 and S4 Figs). The supplementary S1 Table (S1 Table) delineates the correlation coefficient (r) linear regression (LR), signal to noise ratio (s/n) at LLOQ as well as the LLOQ accuracy of our method for simultaneous detection of catechins and theaflavins in the plasma as well as lung and kidney tissue lysates spiked with polyphenols.

The fact that our method was able to achieve a lower limit of quantification of **~** 5 ng/ml in the analysed matrices clearly indicated that it was almost ten to hundred times more sensitive than prevalent HPLC-UV and capillary electrophoresis-based methods [17, 18], and closely comparable to the HPLC-ECD [19] as well as most existing LC/MS-MS protocols [3, 23, 25, 26], notwithstanding deploying polarity switching to accomplish the goal of simultaneous determination of catechins and theaflavins. Notably, no endogenous components were found to significantly interfere with the analytes around their recorded retention times establishing the high selectivity of our method.

Using the chromatographic conditions described above, the retention times for elution were uniformly observed to be 3.02 ± 0.035 min for EGC, 3.70 ± 0.029 min for EC, 3.78 ± 0.032 min for EGCG, 4.48 ± 0.027 min for ECG, 5.74 ± 0.057 min for TF, 6.22 ± 0.049 min for TF3G, 6.48 ± 0.048 min for TF33'diG and 6.62 ± 0.053 min for TF3'G for all the analysed matrices. Compared to previously reported HPLC-UV protocols [16–18] or the single reported HPLC-ECD method [19] our procedure was up to seven times faster in accomplishing complete separation of the individual polyphenols. In fact, our method was also faster or comparable to the existing LC/MS-MS protocols used for such analyses even though such methods have been largely limited to the detection of catechins alone [23, 25].

### Simultaneous Determination of Catechins and Theaflavins in Pure Black Tea Infusion as well as Plasma, Lung and Kidney of Tea Consuming Animals

The major objective of our study was to develop a broadly applicable, rapid and sensitive method for simultaneous detection of catechins and theaflavins across different matrices. To examine whether we can accomplish this task with our developed method, we applied it to pure black tea infusions as well as different tissues of tea-consuming guinea pigs, and found that it could indeed simultaneously detect catechins and theaflavins in most of the analyzed samples. Our method successfully assessed the relative content of such polyphenols in 5% ABT infusion as 51.5 ± 6.2 μg/ml EGC, 56.3 ± 5.5 μg/ml EC, 310.8 ± 10.5 μg/ml EGCG, 104.7 ± 8.8 μg/ml ECG, 30.3 ± 5.1 μg/ml TF, 54.8 ± 5.2 μg/ml TF3G, 37.1 ± 4.1 μg/ml, TF3'G, 125.9 ± 10.2 μg/ml TF33'diG compared to 5% DBT infusion which had 75.3 ± 9.4 μg/ml EGC, 91.6 ± 11.8 μg/ml EC, 893.2 ± 25.2 μg/ml EGCG, 210.3 ± 11.0 μg/ml ECG, 22.6 ± 4.1 μg/ml TF, 15.2 ± 5.0 μg/ml TF3G, 11.5 ± 4.1 μg/ml TF3'G and 28.6 ± 5.1μg/ml TF33'diG (Table 2, S2 Fig). The catechin content of the 5% DBT infusion was therefore found to be over two-fold higher than that of 5% ABT infusion, while the latter had significantly higher levels of total theaflavins (P < 0.05) compared to DBT (S2 Fig). Such observation was also in agreement with earlier reports [31, 32]. Consequently, DBT-treated animals received significantly higher amounts of catechins in comparison to their ABT-administered counterparts who in turn received relatively higher amounts of theaflavins compared to their DBT-treated counterparts. This was indeed reflected in the relative content of these polyphenols detected in the plasma, lung and kidney tissues of the ABT and DBT treated animals (Tables 3 and 4).

**Table 2:** Bioactive Polyphenol Content of Assam and Darjeeling Black Tea infusion^1^^,^^2^.

Tea Infusion	Catechin Content (μg/ml)				Theaflavin Content (μg/ml)			
	EGC	EC	EGCG	ECG	TF	TF3G	TF3′G	TF33′diG
**Assam Black Tea**	51.5 ± 6.2^a^	56.3 ± 5.5^b^	310.8 ± 10.5^c^	104.7 ± 8.8 ^d^	30.3 ± 5.1	54.8 ± 5.2^e^	37.1 ± 4.1^f^	125.9±10.2^g^
**Darjeeling Black Tea**	75.3 ± 9.4^a^	91.6 ± 11.8^b^	893.2 ± 25.2^c^	210.3 ± 11.0^d^	22.6 ± 4.1	15.2 ± 5.0^e^	11.5 ± 4.1^f^	28.6 ±5.1^g^

EGC, Epigallocatechin; EC, Epicatechin; EGCG, Epigallocatechin-3-gallate; ECG, Epicatechin-3-gallate; TF, Theaflavin; TF3G, Theaflavin-3-monogallate; TF3'G, Theaflavin-3'-monogallate; TF33'diG, Theaflavin-3,3'-digallate

^1^ 5% tea infusion brewed for 5 min with continuous stirring

^2^ Values are means ± SD, n = 3

^a,b,c,d,e,f,g^ Items with the same superscript notation in the same column are significantly different from each other as analyzed by the paired t-test (P < 0.05).

**Table 3:** Levels of Tea Polyphenols Detected in Guinea Pig Plasma after Oral Administration of ABT and DBT^1^^,^^2^

Analyte	DBT Treated (ng/ml)	ABT Treated (ng/ml)
Epigallocatechin (EGC)	12.9 ± 5.2	8.4 ±6.9
Epicatechin (EC)	11.6 ± 11.6^3^	7.6 ± 4.6^3^
Epigallocatechin Gallate (EGCG)	130.8 ± 22.0^a^	40.2 ± 5.2^a^
Epicatechin Gallate (ECG)	34.6 ± 4.1^b^	19.3 ± 7.2^b^
Theaflavin (TF)	nd	5.6 ± 1.9^3^
Theaflavin-3-gallate (TF3G)	nd	4.1 ± 2.3^3^
Theaflavin-3′-Gallate (TF3′G)	nd	3.4 ± 1.9^3^
Theaflavin33′diGallate (TF33′G)	nd	5.2 ± 3.8^3^

Quantifiable polyphenol levels in the plasma of ABT and DBT treated animals were significantly greater compared to the untreated counterparts (P < 0.05).

^1^nd, not detected

^2^Values are means ± SD, n = 6

^3^Could not be accurately quantified since the indicated analyte response was detected below the quantification limit in some (upto 50%) of the analyzed samples.

^a,b^ Items with the same superscript notation in the same row are significantly different from each other as analyzed by paired t-test. (P < 0.05).

**Table 4:** Levels of Tea Polyphenols Detected in Guinea Pig Lung and Kidney after Oral Administration of ABT or DBT^1^.

Analyte	Lung		Kidney	
	5% DBT Treated (ng/gm)	5% ABT Treated (ng/gm)	5% DBT Treated (ng/gm)	5% ABT Treated (ng/gm)
**Epigallocatechin (EGC)**	35.8 ± 6.3	28.6 ± 5.9^2^	49.1 ± 17.1	34.2 ± 8.2
**Epicatechin (EC)**	36.6 ± 6.8^2^	15.4 ± 6.7^2^	47.8 ± 11.0	22.3 ± 9.7^2^
**Epigallocatechin Gallate (EGCG)**	144.6 ± 12.5^a^^,^^b^	90 ± 9.5^a^^,^^c^	91.8 ± 15.2^b^^,^^d^	45 ± 7.7^c^^,^^d^
**Epicatechin Gallate (ECG)**	121.8 ± 20.3^e^^,^^f^	58 ± 6.7^e^^,^^g^	22.5 ± 4.7^f^	12.85 ± 2.7^2^^,^^g^

Assessed levels of none of the detected theaflavins were above the LLOQ and therefore were not quantifiable.

The levels of all the quantifiable polyphenols in the lung and kidney of both the ABT and DBT-treated animals were significantly higher than their untreated counterparts (P < 0.05).

^1^Values are means ± SD, n = 6

^2^Could not be accurately quantified since the indicated analyte response was detected below the quantification limit in some (up to 50%) of the analyzed samples.

^a,b,c,d,e,f,g^ Items with the same superscript notation in the same row are significantly different from each other as analyzed by paired t test. (P < 0.05).

Oral administration of both 5% ABT and DBT black tea infusion to guinea pigs for 14 days resulted in detectable accumulation of all major polyphenols in the plasma of the treated animals as evidenced through our UPLC-MS/MS analysis (Table 3 & S3 Fig). The mean level of EGC in the plasma of the 5% DBT-treated animals was found to be approximately 12.9 ng/ml while that of EC, EGCG and ECG, 11.6 ng/ml, 130.8 ng/ml and 34.6 ng/ml respectively (Table 3). Plasma of animals consuming 5% ABT contained approximately 8.4 ng/ml EGC, 7.6 ng/ml EC, 40.2 ng/ml EGCG and 19.3 ng/ml ECG (Table 3). As expected, relative levels of plasma catechins were more in the guinea pigs administered DBT infusion compared to the ABT infusion administered animals. Although, the plasma content of individual catechins was found to increase with increasing levels of catechins in the administered black tea, such dose-dependent increase was found to be statistically significant only for the gallated catechins, namely, EGCG and ECG (P < 0.05) (Table 3). Apparently due to the low abundance of theflavins in DBT, we were unable to detect these polyphenols in the plasma of the DBT-treated animals (Table 3 & S3C Fig). However, we could detect modest levels of all the theaflavins in the plasma of the ABT-treated animals, using our method (Table 3 & S3B Fig). Whether this difference in the quantified levels of plasma catechins and theaflavins (Table 3) is attributable only to the lower abundance of theaflavins compared to catechins in black tea (Table 2), or also to a possible difference in bioavailability or bio-retention of the two polyphenols, needs to be further investigated.

While the plasma levels of the polyphenols help to provide valuable insights on their relative rates of absorption *in vivo*, it does not necessarily furnish accurate information on their precise organ distribution. Considering the projected health benefits of these polyphenols in various human diseases, it is imperative to determine their distribution in the organs involved in such diseases. Thus our present animal study is a step forward towards the future elucidation of the accurate tissue levels as well as the possible protective role of these polyphenols in such diseases.

The polyphenol content in the lung and kidney tissues of the ABT and DBT-treated animals is summarised in Table 4. Catechin levels in both the lung and kidney tissues of the DBT-treated animals were approximately 1.6 times that of the ABT-treated animals (Table 4). This can be perhaps explained by the relatively higher catechin content of the DBT infusion compared to the ABT infusion (Table 2). The total catechin level in the lung was significantly higher (P < 0.05) than the kidney irrespective of whether the animals were administered DBT or ABT infusion (Table 4 & S4 Fig). Although modest levels of theaflavins could be detected both in the lung and kidney tissues in the ABT-treated animals, they could not be quantified as they apparently fell below the LLOQ threshold of our method. Besides the lung and kidney, we also analyzed the liver and heart tissues for their polyphenol content. While free polyphenols (non-metabolised) could not be detected in the liver, low levels of polyphenols were found in the heart tissues. However, such levels were too low (below LLOQ thresholds of our method) for accurate quantification.

While a number of reports have indicated that the peak plasma concentration of polyphenols is achieved within 1–2 hours of consumption of such polyphenols, the exact time taken by them to reach various tissues is still unknown [16, 24]. In our study, we administered the animals with a relatively high dose of 20 ml 5% ABT or DBT infusion per kg body weight in two equal dosages daily for a continuous period of 14 days before evaluating the tissue levels of tea polyphenols. The difference in tissue distribution of the polyphenols observed between different organs in our study could be explained at the level of differential uptake, retention, bioconversion and excretion as is observed with respect to the uptake of any orally administered biochemical including drug molecules. Indeed, differences in vascularisation and blood perfusion, tissue binding, tissue accumulation, local pH, and permeability of cell membranes between the different organs might contribute to the observed dissimilarity in tissue uptake and content of the administered polyphenols. Moreover, after absorption, tea catechins (EC, ECG, EGC, and EGCG) may undergo extensive bioconversion *in vivo* mainly through glucuronidation, sulfation as well as methylation. Such metabolic conversions are already known to be catalyzed by various enzymes like UDP-glucuronosyltransferases (UGT), Catechol-*O*-methyltransferases (COMT), Cytosolic sulfotransferases (SULT) typically present in such tissues [33–37]. As liver is the primary site of such bio-transformation reactions, it is not surprising that we were unable to detect any free polyphenols in this organ. Moreover, both glucuronidation and sulfation have been reported in other tissues like lung and kidney [38–41]. The differential expression and activity of these enzymes in the lung and the kidney tissues may also contribute to the observed disparity in their polyphenol content besides the factors that have been delineated above. The precise factors contributing to our failure to detect these polyphenols in the heart tissues, however mandates a more detailed study. In all, elucidation of the exact factors responsible for the differential content of tea polyphenols in the different organs analyzed in our study is admittedly a complex exercise, and indeed requires to be addressed through a more extensive and carefully designed experimental approach that is beyond the purview of our study, that is primarily dedicated to the development of a method for the simultaneous detection of catechins and theaflavins.

**Fig 2:**
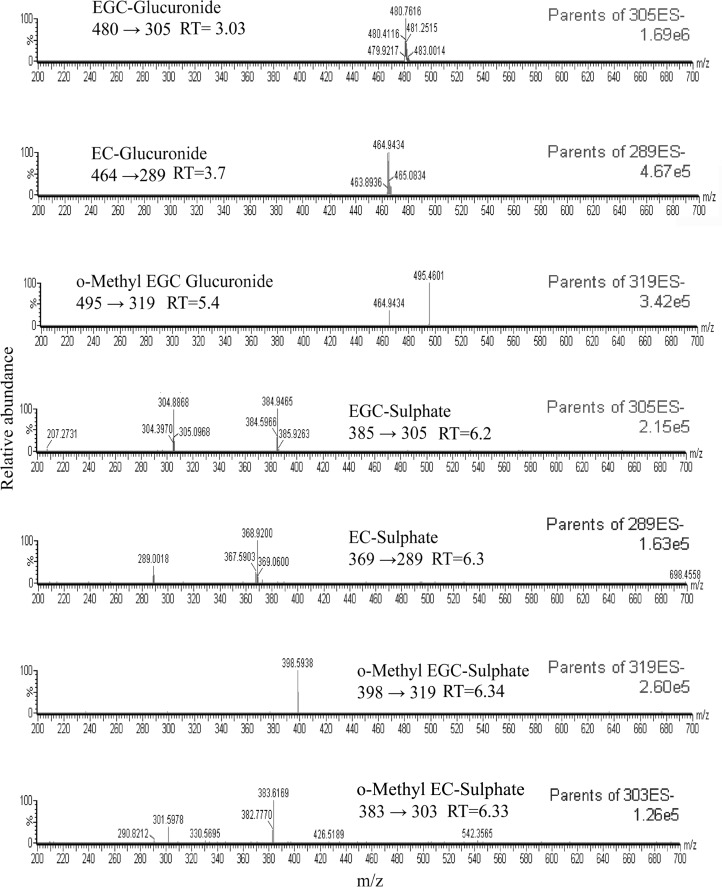
Detection and Identifiction of Catechin Metabolites in the Urine of Tea-Consuming Guinea Pigs. Representative chromatograms for the identification of catechin metabolites in the urine of tea consuming guinea pigs analysed by precursor ion scan showing epigallocatechin glucuronide (EGC-glucuronide), epicatechin glucuronide (EC-glucuronide), o-methyl epigallocatechin glucuronide (o-methyl EGC glucuronide), epigallocatechin sulphate (EGC-sulphate), epicatechin sulphate (EC-sulphate), o-methyl epigallocatechin sulphate (o-methyl EGC-sulphate), o-methyl epicatechin sulphate (o-methyl EC-sulphate) as the primarily identifiable metabolites. The chromatograms also depict the retention times (RT) (highlighted corresponding to the monitoring ions) and the intensity count (highlighted at the right hand side of the chromatograms) of the identified metabolites. Data are representative of three independent experiments done under similar conditions.

Moreover, we strategically did not subject our samples to glucuronidase and sulfatase digestion, as that would have potentially converted the polyphenol metabolites to their free forms. This enabled us to successfully identify the true levels of the biologically active free polyphenols as well as several catechin metabolites in the urine using our protocol. The catechin metabolites were identified using precursor ion scan, by monitoring the negatively charged molecular ions ([M-H]^-^) corresponding to the parent ion *m/z*, daughter ion *m/z* and calculating the associated loss of mass *(m/z)* during fragmentation (S2 Table). After administration of 5% DBT infusion for 14 days, glucuronides and sulfates of EGC and EC were identified in the urine of the treated animals along with the methylated forms of EGC glucuronide, EGC sulfate and EC glucuronide (Fig 2). None of the metabolites of the gallated catechins could however be detected in the urine, consistent with previous reports [20, 27, 42, 43]. This is probably because they are not excreted through the urine as several studies using rat models indicate that EGCG might be instead excreted through the bile [44–46]. This perhaps may also be applicable for our guinea pig model and needs further investigation.

Recognizing the fact that there can be significant variations in the composition of the tea infusions depending on the varieties, sources and conditions of the tea leaves used, as was also observed in our analyses of ABT and DBT infusions (Table 2, S2 Fig), our entire study was carried out with tea leaves from a single lot (single source) of both ABT and DBT. The ABT and DBT used in our study were both purchased from an internationally reputed tea merchant, Goodricke Group Ltd. Evidently, while the DBT infusion was found to be rich in catechins, the tea infusion prepared from ABT was comparatively rich in theaflavins, revealing a significant difference in the composition of these two varieties of black tea (Table 2, S2 Fig). This observation also agrees well with existing reports [31, 32, 47]. A previous study by Henning *et*.*al*., has already shown that variations in flavanol content among tea bags procured from different lots at different times and different stores are smaller compared to differences observed with tea bags representing different brands [48]. However, our primary aim was to develop a method for simultaneous determination of major bioactive tea polyphenols in tea liquor as well as various biological tissues of animals consuming the above two varieties of black tea. Notwithstanding the above-mentioned variations, our method can be effectively utilized for simultaneous detection of different catechins and theaflavins from any source of such polyphenols with high reproducibility.

An important limitation of our study is perhaps the absence of a suitable internal standard which has admittedly restricted proper optimization of the method developed by us for simultaneous detection of catechins and theaflavins. Since an internal standard was not used in our analysis, we had to keep our quantification limits comparatively high, with elevated signal to noise ratio (S1 Table), in order to make adequate provisions for matrix effects and analytical errors. While internal standards for chromatographic analysis of catechins are well characterized, a reliable internal standard is yet to be identified for the analysis of theaflavins. This might partly explain why there are virtually no reported methods for simultaneous detection of catechins and theaflavins with high sensitivity along with the existence of only a few methods for theaflavin analysis compared to the large number of established methods for catechin quantification alone [3, 19]. The use of an internal standard is however not mandatory for relative quantification of polyphenols [49], that could help in the elucidation of the most bio-available and efficacious polyphenol(s) for potential formulation of tea based therapeutic products. Further, our method had an LLOQ of approximately 5 ng/ml, that fell short of allowing dependable quantification of the detected polyphenols in some tissues. This certainly can be circumvented through future improvement of the overall sensitivity of our method. Here again, the non-availability of an internal standard for theaflavins was the main constraint behind our failure to lower the LLOQ of our method to such qualifying levels. In addition, although several catechin metabolites were detected in the urine of the tea consuming animals in our present study, we could not quantify them due to the apparent non-availability of reliable reference standards for the detected metabolites.

It thus needs to be accepted that a method optimization for simultaneous determination of catechins and theaflavins has its own share of challenges and would perhaps take further work to address with due perfection. As our principal aim of the present study was to develop a universal UPLC-MS/MS-based protocol for simultaneous detection of catechins and theaflavins that can be used for all biological matrices, our present method admittedly has the potential to accomplish this goal.

## Conclusion

Overall, we have developed a novel, rapid and sensitive UPLC-MS/MS method for simultaneous detection of major bioactive tea polyphenols, using ESI polarity switching-MS/MS in dual ionization mode, which enables simultaneous detection of catechins and theflavins in pure black tea and tissues of tea consuming guinea pigs. Our present method would certainly allow for rapid and sensitive simultaneous detection of catechins and theaflavins as well as catechin metabolites and can be adapted to a variety of biological matrices as well as natural sources of polyphenols. The method also has provisions for further optimization for detailed pharmacokinetic studies on bioactive polyphenols.

Moreover, our method allows the accurate determination of the relative bioavailability and organ distribution of different tea polyphenols, that would help to assess the true bioefficacy of such natural antioxidants *in vivo*. This in turn would permit prospective use of such natural polyphenols or their high efficacy formulations for prevention of oxidative damage and inflammation in humans for the therapeutic management of various degenerative diseases associated with such pathophysiology.

## Supporting Information

S1 Fig Chemical Structures of Major Catechins and Theaflavins in Black Tea. Chemical structures of the major bioactive tea catechins—epigallocatechin (EGC), epicatechin (EC), epigallocatechin-3-gallate (EGCG), epicatechin-3-gallate (ECG), and theaflavins- theaflavin (TF), theaflavin-3-monogallate (TF3G), theaflavin-3'-monogallate (TF3'G) and theaflavin-3,3'-digallate (TF33'diG) are depicted along with their respective molecular weights (MW).
(TIF)

S2 Fig Bioactive Polyphenol Content of Assam and Darjeeling Black Tea Infusions. Graph showing relative abundance of epigallocatechin (EGC), epicatechin (EC), epigallocatechin-3-gallate (EGCG), epicatechin-3-gallate (ECG), theaflavin (TF), theaflavin-3-monogallate (TF3G), theaflavin-3'-monogallate (TF3'G) and theaflavin-3,3'-digallate (TF33'diG) in 5% Assam Black Tea (ABT) infusion and 5% Darjeeling Black Tea (DBT) infusion as depicted in Table 2. Data were statistically analysed by paired t-test. Significant differences were found between groups as indicated (Asterisks * indicate significant differences at P < 0.05). Data are represented as means ± S.D (error bars) of three independent experiments done under similar conditions.
(TIF)

S3 Fig Levels of Tea Polyphenols Detected in Guinea Pig Plasma Following Oral Administration of ABT and DBT Infusions. Representative MRM chromatograms showing polyphenol levels in the plasma of (A) control guinea pigs as well as guinea pigs orally administered (B) 5% ABT infusion and (C) 5% DBT infusion for 14 days. Samples were collected 120 min after the administration of the final tea infusion (6 hours after the first dose and 2 hours after the second tea dose, on the 14^th^ day of treatment). The relevant names of the analytes are highlighted against their corresponding chromatograms, along with the associated retention times (labelled below the analyte names) and responses (labelled below the retention times). The right-hand top labels on the chromatograms indicate their respective MRM transitions and peak heights. The responses were quantified using the linear regression for plasma given in the S1 Table and presented in the Table 3. Data are representative of at least three independent experiments done under similar conditions with 6 animals per group (n = 6).
(TIF)

S4 Fig Levels of Tea Polyphenols Detected in the Guinea Pig Lung and Kidney Tissues Following Oral Administration of DBT Infusion. Representative MRM chromatograms showing polyphenol levels in (A) control lung tissue lysate, (D) control kidney tissue lysate along with the blank (control) of (B) lung tissue lysate and (E) kidney tissue lysate spiked with polyphenols at their respective LLOQ along with the (C) lung tissue lysate and (F) kidney tissue lysate of guinea pigs administered 5% DBT infusion for 14 days, and collected 2 hours after the administration of final tea infusion (6 hours after the first dose and 2 hours after the second tea dose, on the 14^th^ day of treatment). The relevant names of the analytes are highlighted against their corresponding chromatograms, along with the associated retention times (labelled below the analyte names) and responses (labelled below the retention times). The right-hand top labels on the chromatograms indicate their respective MRM transitions and peak heights. The responses were quantified using the linear regression given in the S1 Table and represented in the Table 4. Data represent three independent experiments done under similar conditions with 6 animals per group (n = 6).
(TIF)

S1 Table Correlation Coefficient (r), Linear Regression (LR), Signal to Noise (s/n) Ratio and LLOQ Accuracy for Different Catechins and Theaflavins in the Plasma as well as Lung and Kidney Tissue Lysates of Control Guinea Pigs Spiked with individual Polyphenols^1,2^. (DOC)

S2 Table Catechin Metabolites Detected in the Urine of Tea Consuming Guinea Pigs and Their Signature Precursor and Product Ions. (DOC)

## Abbreviations

TF: Theaflavin
EGCG: Epigallocatechin-3-gallate
EGC: Epigallocatechin
EC: Epicatechin
ECG: Epicatechin-3-gallate
TF3G: Theaflavin-3-monogallate
TF3'G: Theaflavin-3'-monogallate
TF33'diG or TFdiG: Theaflavin-3,3'-digallate
LC: Liquid Chromatography
MS: Mass Spectrometry
HPLC: High Performance Liquid Chromatography
UPLC: Ultra-Performance Liquid Chromatography
LLOQ: Lower Limit of Quantification
RT: Retention Time
