# Supplementary figures and images for: Simultaneous Determination of Black Tea-Derived Catechins and Theaflavins in Tissues of Tea Consuming Animals Using Ultra-Performance Liquid-Chromatography Tandem Mass Spectrometry
Source: PLoS One. 2016 Oct 3;11(10):e0163498. doi: 10.1371/journal.pone.0163498 (PMC5047449; doi:10.1371/journal.pone.0163498)

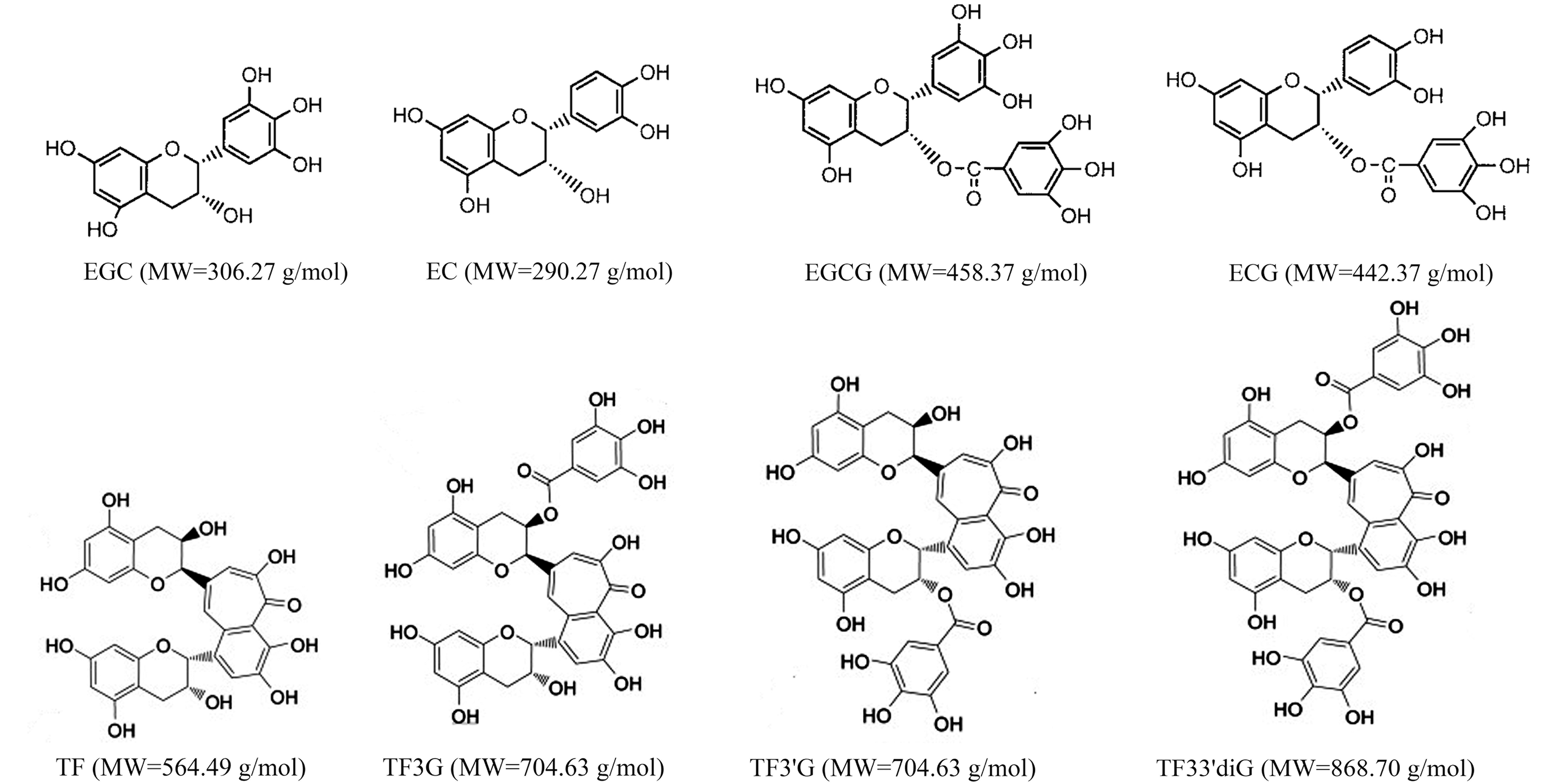

Supplement: S1 Fig — Chemical structures of the major bioactive tea catechins—epigallocatechin (EGC), epicatechin (EC), epigallocatechin-3-gallate (EGCG), epicatechin-3-gallate (ECG), and theaflavins- theaflavin (TF), theaflavin-3-monogallate (TF3G), theaflavin-3'-monogallate (TF3'G) and theaflavin-3,3'-digallate (TF33'diG) are depicted along with their respective molecular weights (MW). (TIF) [file pone.0163498.s001.tif]

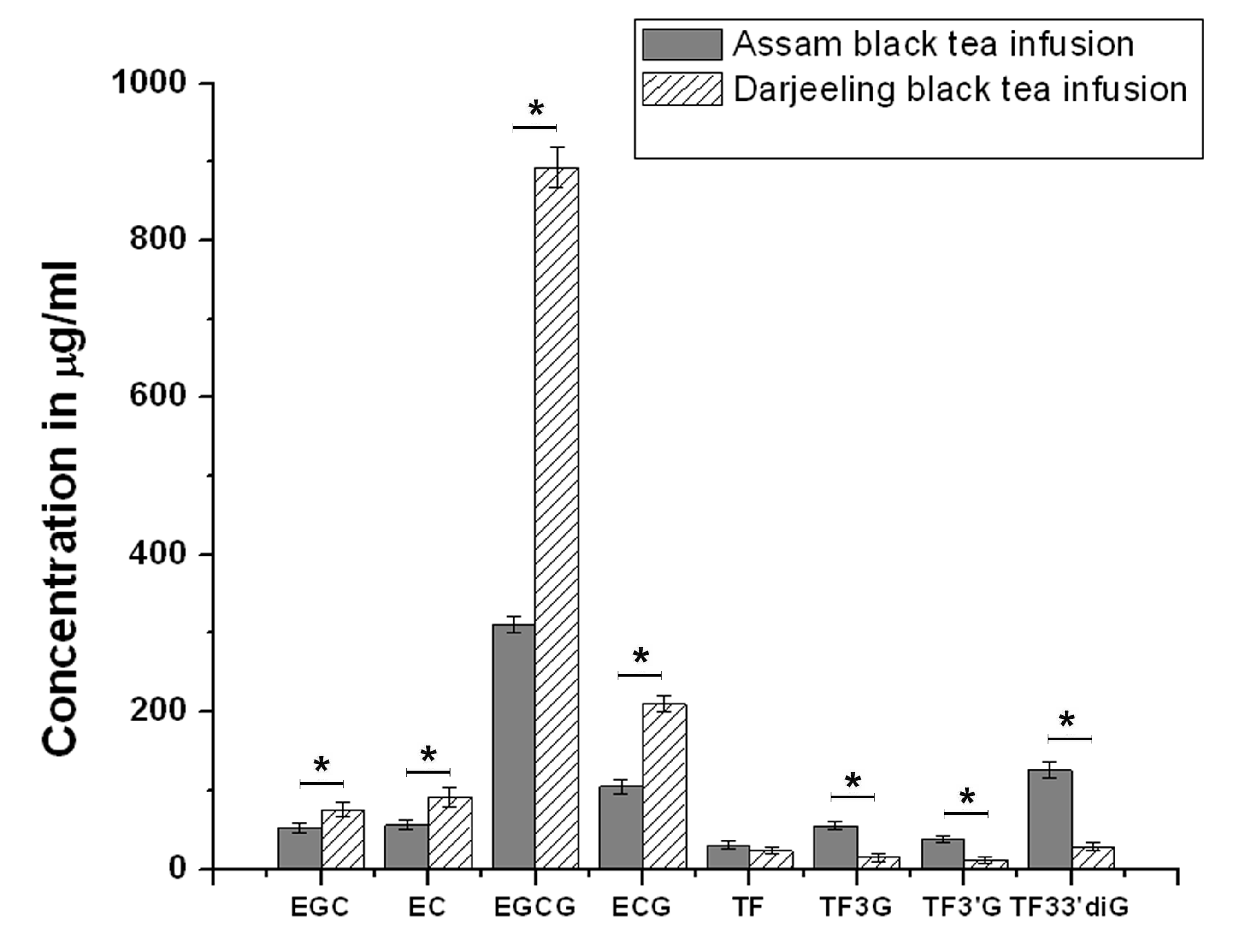

Supplement: S2 Fig — Graph showing relative abundance of epigallocatechin (EGC), epicatechin (EC), epigallocatechin-3-gallate (EGCG), epicatechin-3-gallate (ECG), theaflavin (TF), theaflavin-3-monogallate (TF3G), theaflavin-3'-monogallate (TF3'G) and theaflavin-3,3'-digallate (TF33'diG) in 5% Assam Black Tea (ABT) infusion and 5% Darjeeling Black Tea (DBT) infusion as depicted in Table 2. Data were statistically analysed by paired t-test. Significant differences were found between groups as indicated (Asterisks * indicate significant differences at P < 0.05). Data are represented as means ± S.D (error bars) of three independent experiments done under similar conditions. (TIF) [file pone.0163498.s002.tif]

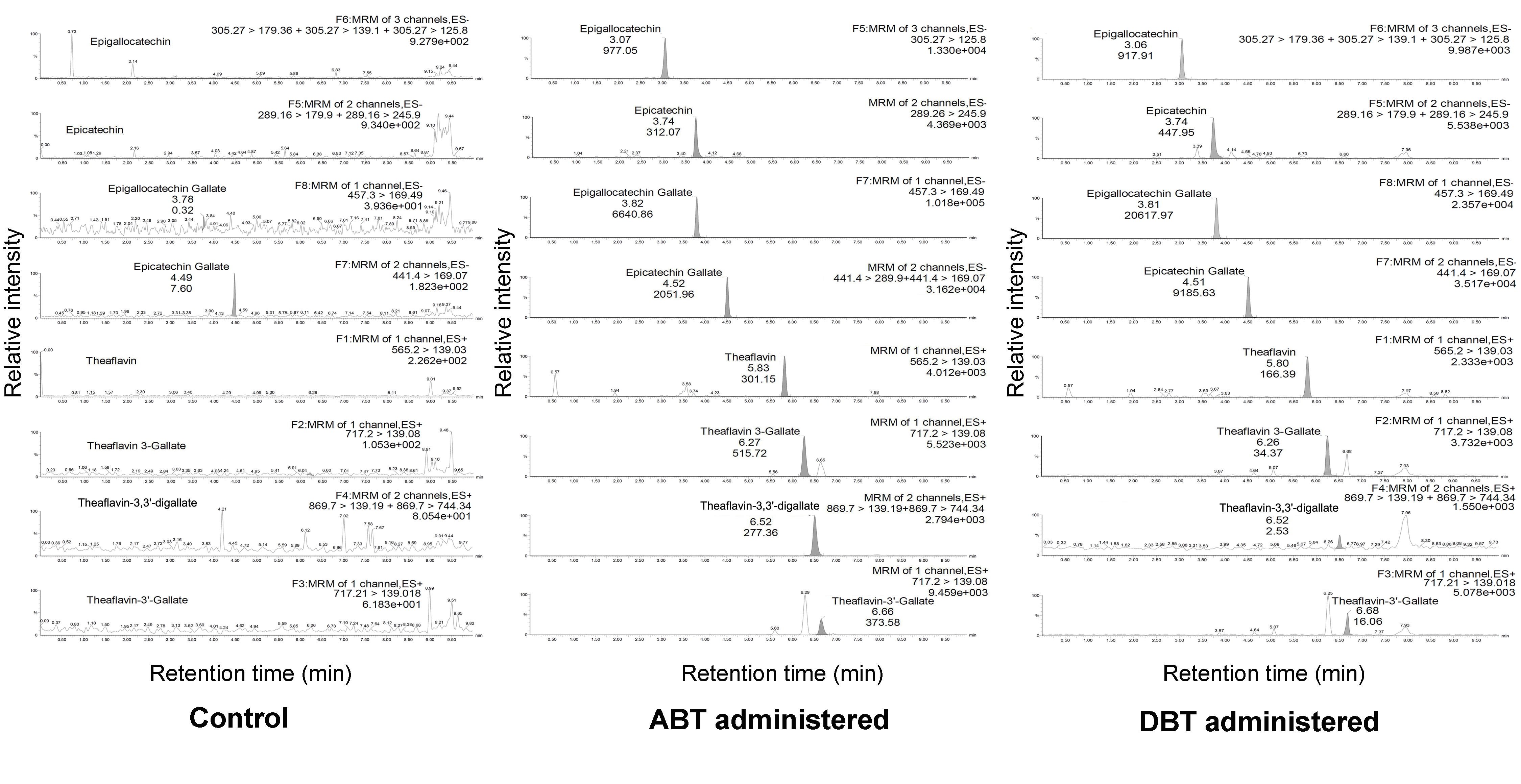

Supplement: S3 Fig — Representative MRM chromatograms showing polyphenol levels in the plasma of (A) control guinea pigs as well as guinea pigs orally administered (B) 5% ABT infusion and (C) 5% DBT infusion for 14 days. Samples were collected 120 min after the administration of the final tea infusion (6 hours after the first dose and 2 hours after the second tea dose, on the 14th day of treatment). The relevant names of the analytes are highlighted against their corresponding chromatograms, along with the associated retention times (labelled below the analyte names) and responses (labelled below the retention times). The right-hand top labels on the chromatograms indicate their respective MRM transitions and peak heights. The responses were quantified using the linear regression for plasma given in the S1 Table and presented in the Table 3. Data are representative of at least three independent experiments done under similar conditions with 6 animals per group (n = 6). (TIF) [file pone.0163498.s003.tif]

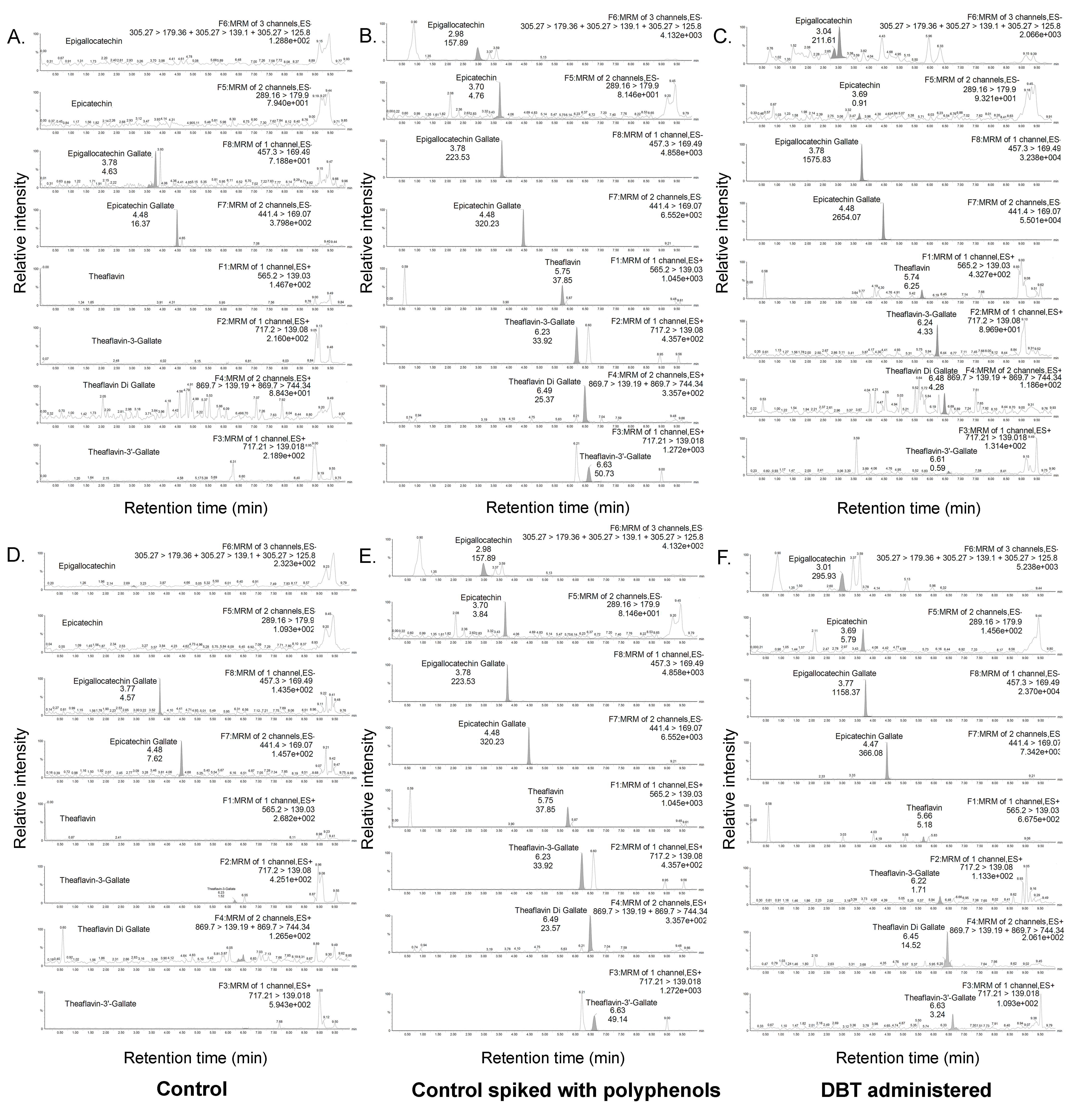

Supplement: S4 Fig — Representative MRM chromatograms showing polyphenol levels in (A) control lung tissue lysate, (D) control kidney tissue lysate along with the blank (control) of (B) lung tissue lysate and (E) kidney tissue lysate spiked with polyphenols at their respective LLOQ along with the (C) lung tissue lysate and (F) kidney tissue lysate of guinea pigs administered 5% DBT infusion for 14 days, and collected 2 hours after the administration of final tea infusion (6 hours after the first dose and 2 hours after the second tea dose, on the 14th day of treatment). The relevant names of the analytes are highlighted against their corresponding chromatograms, along with the associated retention times (labelled below the analyte names) and responses (labelled below the retention times). The right-hand top labels on the chromatograms indicate their respective MRM transitions and peak heights. The responses were quantified using the linear regression given in the S1 Table and represented in the Table 4. Data represent three independent experiments done under similar conditions with 6 animals per group (n = 6). (TIF) [file pone.0163498.s004.tif]
