# Supplementary material for: Simultaneous Determination of Black Tea-Derived Catechins and Theaflavins in Tissues of Tea Consuming Animals Using Ultra-Performance Liquid-Chromatography Tandem Mass Spectrometry
Source: PLoS One. 2016 Oct 3;11(10):e0163498. doi: 10.1371/journal.pone.0163498 (PMC5047449; doi:10.1371/journal.pone.0163498)
Supplement: S1 Table — (DOC) [file pone.0163498.s005.doc]

**S1 Table. Correlation Coefficient (r), Linear Regression (LR), Signal to Noise (s/n) Ratio and LLOQ Accuracy for Different Catechins and Theaflavins in the Plasma as well as Lung and Kidney Tissue Lysates of Control Guinea Pigs Spiked with individual Polyphenols^1,2^.**

| **Matrix** | **Polyphenol** | **Correlation Coefficient** | **Linear Regression**^3^ | **Signal:Noise**  **at LLOQ**^4^ | **LLOQ**^4^ **Accuracy** |
| --- | --- | --- | --- | --- | --- |
| **Plasma** | EGC | 0.9950 | Y = 35.41x + 165.33 | 86.28 | 106.77 |
|  | EC | 0.9920 | Y = 6.87x + 205.37 | 42.76 | 99.25 |
|  | EGCG | 0.9976 | Y =134.98x + 330.11 | 2043.35 | 109.89 |
|  | ECG | 0.9968 | Y =196.93x + 632.15 | 3941.18 | 108.15 |
|  | TF | 0.9984 | Y = 30.99x + 14.98 | 321.67 | 101.96 |
|  | TF 3G | 0.9971 | Y = 45.31x + 153.71 | 915.06 | 101.22 |
|  | TF 3'G | 0.9983 | Y = 35.26x + 158.01 | 145.55 | 108.70 |
|  | TF 33'diG | 0.9986 | Y =18.03x + 55.21 | 313.02 | 93.40 |
| **Lung** | EGC | 0.9997 | Y =13.5x + 91.77 | 16.36 | 97.83 |
|  | EC | 0.9753 | Y = 0.61x − 0.98 | 13.75 | 93.36 |
|  | EGCG | 0.9983 | Y = 49.8x − 33.35 | 490.04 | 103.17 |
|  | ECG | 0.9960 | Y = 76x − 41.64 | 99.79 | 95.24 |
|  | TF | 0.9985 | Y = 10.17x −11.91 | 42.96 | 96.68 |
|  | TF 3G | 0.9986 | Y = 12.01x − 20.13 | 80.47 | 89.68 |
|  | TF 3'G | 0.9939 | Y = 8.08x + 15.09 | 53.68 | 97.41 |
|  | TF 33'diG | 0.9978 | Y = 4.5x + 5.22 | 38.04 | 96.60 |
| **Kidney** | EGC | 0.9961 | Y = 13.6x + 94.30 | 16.59 | 93.81 |
|  | EC | 0.9948 | Y = 0.6x − 2.25 | 52.22 | 102.62 |
|  | EGCG | 0.9983 | Y = 49.7x − 19.47 | 522.23 | 97.80 |
|  | ECG | 0.9985 | Y = 73.20x − 35.38 | 101.03 | 97.22 |
|  | TF | 0.9999 | Y = 10.53x −16.77 | 42.34 | 103.22 |
|  | TF 3G | 0.9996 | Y = 15.33x − 39.70 | 78.21 | 96.81 |
|  | TF 3'G | 0.9993 | Y = 11.26x − 4.50 | 84.69 | 104.20 |
|  | TF 33'diG | 0.9977 | Y = 6.52x − 7.69 | 31.23 | 105.61 |

EGC: Epigallocatechin; EC: Epicatechin; EGCG: Epigallocatechin-3-gallate; ECG: Epicatechin-3-gallate; TF: Theaflavin; TF3G: Theaflavin-3-monogallate; TF3'G: Theaflavin-3'-monogallate; TF33'diG or TFdiG: Theaflavin-3,3'-digallate

**^1^**Plasma and tissue lysates of lung and kidney from control (untreated) guinea pigs were spiked with polyphenol standards, to final concentrations in the range of 5 to 320 ng/ml (except EC, that had a range of 10-640 ng/ml).

^2^Data are representative of three independent experiments done under similar conditions.

^3^*y* =analyte response; x=concentration in ng/ml;

^4^LLOQ = Lower limit of quantification of approximately 5 ng/ml for all polyphenols
