# Supplementary material for: Simultaneous Determination of Black Tea-Derived Catechins and Theaflavins in Tissues of Tea Consuming Animals Using Ultra-Performance Liquid-Chromatography Tandem Mass Spectrometry
Source: PLoS One. 2016 Oct 3;11(10):e0163498. doi: 10.1371/journal.pone.0163498 (PMC5047449; doi:10.1371/journal.pone.0163498)
Supplement: S2 Table — (DOC) [file pone.0163498.s006.doc]

| S2 Table. Catechin Metabolites Detected in the Urine of Tea Consuming Guinea Pigs and Their Signature Precursor and Product Ions | | | |
| --- | --- | --- | --- |
| Metabolites | Precursor Ion | Product Ion | Ion loss |
| Epicatechin glucuronide (EC-Glu) | 465 | 289 | 176 |
| Epicatechin sulfate (EC-S) | 369 | 289 | 80 |
| *O*-methyl-Epicatechin sulphate (m-EC-S) | 383 | 303 | 80 |
| Epigallocatechin glucuronide (EGC-Glu) | 481 | 305 | 176 |
| Epigallocatechin sulfate (EGC-S) | 385 | 305 | 80 |
| *O*-methyl-Epigallocatechin glucuronide (m-EGC-glu) | 495 | 319 | 176 |
| *O*-methyl-Epigallocatechin sulfate (m-EGC-S) | 399 | 319 | 80 |
